# Extra Supplement—The Nursing Mirror

**Published:** 1890-04-19

**Authors:** 


### "The Hospital" Nursing Mirror.

Being the Extra Nursing Supplement of "The Hospital" Newspaper.

All Contributions for this Supplement should be addressed to the Editor, The Hospital, 140, Strand, London, W.C., and should have the word "Nursing" plainly written in left-hand top corner of the envelope.

### En Passant.

THOUSANDS OF POUNDS FOR THE NATIONAL PENSION FUND.—We have the pleasure to state that Messrs. Glyn, Mills, Currie, and Co. have promised to give £500, providing the other bankers will subscribe the remaining £1,500 required to at once bring the Donation Bonus Fund up to £40,000. Contributions of any amount may be sent to the Editor of The Hospital, or to the Manager, National Pension Fund for Nurses, 8, King Street, Cheapside, London, E.C.

Amount already acknowledged, **Twelve Thousand Pounds.**

SOUTH WIMBLEDON.—The first annual meeting of subscribers to the South Wimbledon District Nursing Association was held last month, to receive a report. The nurse, Miss Plumb, entered upon her duties early in April, 1889. At the outset the work was, as is the case in all new institutions, hardly understood by those interested, but as its great merits became known, it rapidly increased, and is now quite sufficient to engage the whole attention of at least one nurse. The medical men in the district have materially aided the association by employing the nurse in many urgent cases, and they all bear testimony to the comfort she has brought to those unable to afford the services of paid nurses. The number of cases treated by the nurse has been 94, and the total number of visits paid has been 1,879. Miss Plumb, owing to failing health, has tendered her resignation, and the association must seek the services of another nurse.

NURSING IN PARIS.—A petition in favour of the readmission of the Sisters of Charity to the hospitals has been signed by nine of the retiring municipal councillors —MM. Ferdinand Duval, F. Riant, Denys Cochin, Gamard, Lerolle, Dufaure, Despatys, Deville, and George Berry. The following is the principal passage:—"These admirable servants of the poor have been expelled without taking into consideration either the objections of the sick or the protests of the doctors. The consequence has been to deprive the labouring population, who are too needy to obtain it in their own houses, of the services of those nurses whom the rich, without distinction of creed, call in as soon as they are attacked by disease. In asking you to restore to the Sisters the control of the services which was taken from them, we have no political object. We have simply at heart the interest of the sick. If you receive our petition favourably, you will give satisfaction to the great majority of the Parisian population."

DR. PAGE AT REDDITCH.—In the beginning of the month Dr. Page gave a public lecture on "Home Nursing" at Redditch. He said that nursing was divided into two branches—professional and non-professional or domestic. It was the latter they were to consider. There was good and bad nursing. Five and twenty years ago the typical nurse was a person advanced in life, not over clean, and decidedly addicted to the use of stimulants; but the Mrs. Gamp description of nurse was fast going out. Of all the occupations open to women, none were of a nature more suited to the sex. It was an occupation requiring acuteness, delicacy of touch, and tenderness of heart, and in all these qualities women excelled, and were thus calculated to render most grateful help to the genus homo. Men, however, if properly trained, might make good nurses, and they were required in certain conditions of life, as in the army, workhouses, and asylums. A good nurse should possess presence of mind, gentleness, should be accurate, should have a good memory, good observation and forethought. Some of Dr. Page's remarks seem to have suffered at the hands of the reporters, but the lecture must have been both practical and interesting.

SIR HENRY ACLAND ON NURSES.—Sir Henry Acland lately addressed the supporters of the Banbury Nursing Association. Speaking of the district nurse, he said she required to be more useful and more capable than a nurse in a hospital, because she was more self-dependent in a district—where perhaps she rarely ever saw the medical man—than her sister in the well-regulated system of a hospital, who was, as it were, surrounded by assistants. What a nurse ought to be was a question of great importance, and he could best describe her by using the words of Florence Nightingale, who considered she should possess, amongst other attractions, which he read, the power of being a living "Sermon on the Mount." According to his own thinking, a really good nurse was more like an angel in character and conduct than any person he could see on earth. It is nice to know Sir Henry has such a high ideal for nurses, but there are some nurses who are good, useful creatures, and not angels, who are hampered by the tremendous height which the public expect them to strive to reach.

QUEEN VICTORIA'S JUBILEE INSTITUTE.—We have received the conditions of affiliation with the above Institute, from which we quote the following:—"The following qualifications shall be considered requisite in order to entitle a nurse to be placed on the roll of Queen's Nurses, namely:—(*a*) Training at some approved General Hospital or Infirmary for not less than one year. (*b*) Approved training in district nursing for not less than six months, including the nursing of mothers and their infants after childbirth. (*c*) Nurses in country districts (in accordance with par 8 of these conditions) must have at least three months' approved training in midwifery. N.B.—These conditions are subject to modification by the Council, to meet exceptional cases of those now engaged in district work who may afford satisfactory evidence of competency. Besides training district nurses, the Committee will, when their funds permit, aid the establishing of new District Homes." We are glad to see that Miss Rosalind Paget, a trained nurse and midwife, is appointed General Inspector of Nursing.

A SUGGESTION FOR A MEMORIAL.—It has been suggested to us that an appropriate memorial to the late Mr. Junius S. Morgan, would take the form of a Benevolent Branch of the National Pension Fund. The Provident branch of this same Fund is now fully provided for, but there are ever occurring cases of nurses who, through no fault of their own, have fallen upon evil days, and it was just such cases Mr. Morgan delighted to help. The Provident branch of the fund owes its prosperity chiefly to Mr. Morgan, and surely it would be a gracious act on the part of those who will benefit chiefly by Mr. Morgan's generosity, if they try in their turn to benefit their less fortunate sisters in the name of their own benefactor? This might be done if all, or most of the first thousand would try between now and the end of June to collect a purse of money to be presented to the Princess of Wales to form a nucleus for a benevolent fund to be called the "Junius S. Morgan Memorial." The articles of association of the National Pension Fund include, as one of the objects of the society, the making of *ex gratia* payments or gifts to nurses not being members of the society, and now the £40,000 is practically raised for the bonus fund, it is time the benevolent branch was brought forward. If the nurses could collect on an average a sum of £5 it would prove their gratitude to Mr. Morgan in a peculiarly appropriate way, and show that as a body they are ready to help one another, and to combine to perpetuate a good man's memory.

### Nursing Lectures.

By Percy Lewis, M.D.

### Lecture VIII.—FEEDING.

It is in feeding patients that the difference between trained and untrained nurses comes out so markedly. Nurses can contribute in a very large manner to a patient's welfare in this matter, for the way in which food is given makes all the difference as to whether a patient takes it or not. If food is given in an untidy fashion the first glance will set the patient against it; it should be in the middle of the plate and not allowed to soil it all over, nor should a meal be taken to the patient until everything is ready for him to begin; he should not be kept waiting for bread or knife or spoon,nor should he be given more than enough. You have all perhaps had a feeling of disgust excited when being a little out of sorts you come to dinner and get a big helping. How often have you gone away without eating when you would have finished a smaller portion easily? It is the same with patients. Never keep food in the sick-room or ward, nor, as much as possible, allow patients to see food which they may not have. Meals should be given most punctually, as much as is ordered and nothing more. Miss Nightingale says that patients frequently only manage to get their appetites up for certain hours of the day, generally when they expect their meals, and that a few minutes waiting will destroy it. Never talk to patients at their meals or allow anything to go on in the wards which would detract their attention from their food. Food should never be left by a patient's side, in case he should feel inclined to take it. This is a custom against which Miss Nightingale has entered a most emphatic protest. Should a patient express a wish for any special article of diet, remember to ask the doctor at the next visit. Cold water may be generally given in small quantities at a time, but thirst will be best relieved by a teaspoonful of cold coffee or a slice of lemon. Perhaps, however, the best prevention of thirst is to keep the mouth scrupulously clean. If necessary, ask the doctor for a mouth-wash; the glycerine of borax, commonly used, is from its sweet taste often disagreeable. Ice is useful, but makes the patient more thirsty after, and occasionally causes flatulence. It should be kept at the patient's side in a vessel on the soap-dish principle, so that the water may drain off as it melts. The practice of keeping it on flannel has rather a tendency to make the ice taste of the flannel or of the soap with which it was washed. The best way to break ice is to split off small pieces with a large pin. It is best always to have instructions as to whether to wake a patient for food. If you have had none it is left to your judgment. Should the case be one which does not get enough sleep, don't wake him; on the other hand you need have no scruples in waking a patient who is a chronic case and sleeps well at night. Some nurses only wake their patients partially, just sufficiently to take their food, after which they quickly fall asleep again. This is a great accomplishment, and one well worth learning.

Now, I told you that foods were divided into sugars, albumens, fats, extractives, salts, and water; the body is also composed of the same materials, and as the body is always wasting, hence the necessity for these substances to be constantly taken in as food. A perfect article of diet would therefore be one which contained all these substances, in fact one would be able to live on such a substance. Does such a thing exist? Most of us have lived our first nine months on such a diet, viz., milk. Milk contains all the materials necessary for life, and in a liquid, easily digestible state. Many adults suffering from kidney and other diseases live for several months and grow fat on milk alone, feeling all the time in the best of health. I have laid special stress on the nutritive value of milk, because patients often think that they are being starved when kept on this diet, and a sympathetic nurse, unless she understands this point is apt to agree with them. Milk forms the diet of babies because their digestions are too feeble to stand anything else. For the same reason milk forms the chief diet in most acute diseases, for in them the digestion is always more or less out of order. The state of the tongue gives us the clue as to how it is affected, which is the reason why we look at it. The gastric juice is an acid juice. The effect of adding an acid such as vinegar or lemon juice to milk is to form curds. Cows' milk forms very large curds in the stomach which are very indigestible. Small digestible curds are substituted for large ones by boiling the milk or diluting it with soda water, in one of which forms it is generally given to the sick. Another way of making milk digestible is to pre-digest it before it is swallowed by adding different digestants to it, such as pepsin, liquor pepticus, Benger's food; the latter is a starchy food which has the digestant mixed with it.

In hospitals we have a diet table drawn up for convenience in ordering and in making up the accounts. The diets are so arranged that they form a sliding scale from fever diets, through soup, farinaceous, and fish diets up to ordinary diets, starting with nothing but milk, and approaching nearer and nearer to health diet until full diet is reached. They are arranged in meals at stated times with the exception of fever diets. These latter must be spread out over the twenty-four hours—so much, so often. For instance, fever diet No. 1., consisting of three pints of milk, does not mean three meals of a pint each, but two and a half ounces every hour. The object of cooking food is to make it more digestible; and although in hospitals the cooking is all done for you, yet every nurse should be a bit of a cook as every surgeon should be a bit of a carpenter. If you get a chance of learning a little invalid cooking, don't throw the chance away. The public, you will find, have great faith in jelly, so that it is as well for you to know that it is almost of no use as a food. It has been found by experiment that animals fed on jelly alone died just as soon as animals not fed at all. When made with milk it is only of as much value as the milk it contains.

Another popular delusion exists as to the value of beef-tea. With reference to this subject I am accustomed to quote from the late Dr. Fothergill's book on dietetics. This is what he says: "Beef tea is not a food, it is a stimulant," "to give beef-tea alone to a sick person, is to give him a stone when he asks for bread. Grateful and acceptable to the palate and stomach, possessing stimulating properties, beef-tea has its value. But all the same as regards its food value it is but a jackass in a lion's skin."

Stimulants are not foods, but are substances which enable us to make use of energy stored up in our bodies in the shape of fat, muscle, &c. They enable us in fact to feed upon ourselves. As it is obvious that this process could not go on long, they are chiefly used temporarily to tide us over a critical period in an acute disease, or to stimulate digestion in a chronic one. Alcohol is not now used to the large extent formerly the practice and which still lingers in some hospitals. But alcohol is not the only stimulant; beef tea we have just heard is one, so are tea and coffee, and extracts of meat such as Leibig's. Stimulants are given in small quantities at a time and at such intervals that the second dose is given before the effect of the previous one has worn off, so you see at once the importance of the most absolute punctuality.

Dieting is a complicated subject. Nurses think doctors insist on this or withhold that article of diet out of mere caprice. I assure you it is nothing of the sort. Each substance is considered not only as to its digestibility, but also as to its complex chemical composition. Your part of dieting consists in a most loyal carrying out of the instructions you receive from the doctor in charge of the case.

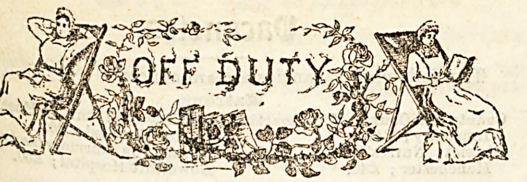

### The Passing of the Shadow.

Prologue.

Frank and I were sitting over our dessert. The lamp lighted up everything in its own circle with clear-cut exactitude; while without the circle, the room was obscured by a brown mistiness. There was a conscious affectation in the turn of the maidenhair fern in the specimen glass; the great round oranges seemed basking in the light in quiet content; the broken nutshells lay in what appeared to be studied picturesqueness on the dessert plates; the glasses stood out in strangely defined precision, while the wine "trembled and sparkled as in ecstasy."

"This is a snug little retreat you have made for yourself," said my friend, after a break in the conversation; "the rich brown of your walls gives it a particularly cosy flavour."

"Yes," replied I, pouring him out a glass of claret, "believing that my thoughts are inclined to be flighty, I paint my walls brown, that my character may assume a decorous and sober hue."

"Another of your whims, you dear old fellow," said he, "as if colour could influence the moral qualities!"

"And why not?" I said, leaning back comfortably in my chair, "the face is but an index to the mind, and you agree that colour influences the face! Look at those who live in the country! The light of the buttercups and roses is reflected on their cheeks, while you catch flashes of the yellow sun in their smiles; on the contrary, the complexions of those who live in the narrow gloomy streets have caught the pale ashen grey colour of what is round them, and again—"

"You mistake," interrupted he; "It is the circumstances in which they live, not the colour that influences their looks."

"Not altogether," said I. "I was staying in an old chateau in France, in which the rooms took their names from the colour most prominent in their decoration. Each of the three daughters had chosen a room painted in her favourite colour, light green, pale yellow, and red respectively; and I can assure you that their characters partook of the character of the colouring. The 'green' maiden was fresh, gentle, full of poetry and the love of nature, with a wild wayward grace about her; the 'yellow' maiden, though quiet and self-absorbed, was full of suppressed yet twinkling merriment, which sometimes burst forth into brilliant flashes of wit; the 'red' maiden was proud, haughty, passionate, queenlike. And remember that these girls were all brought up under the same circumstances in a retired part of France."

"The dream of a dreamer," said my friend; but I saw he was attending.

"An interesting little story is connected with them," said I, "which I heard when over there, and dotted roughly down. You shall have it to read if you like."

I.

Without, the sun was setting, but night had already begun to lurk in the folds of the dark rich red draperies, which trailed in an overgrowth of luxuriance on the carpeted floor. Through the red blind came a misty light, which the mirror, also heavily draped, quite failed to reflect, and caught instead at a pure line of sun through the half-closed door. This light it reproduced with almost exaggerated brilliancy, and it shone like a thin line of jewels from the frame. It might have been a tropical forest, for the air was oppressively close, and overladen with perfume; while no deep growth of moss could have been softer than the carpets and cushions; no festoons of creepers thicker than the dark curtains. And leaning back on a couch, breathing this air of dense magnificence, was a young girl, clad in a loose red dress. Her face was oval-shaped and rather pale, but there was more colour on the lips and cheeks than was caught from the rich hangings, and more light in the eyes than was reflected from the iridescent beads with which she was toying. Her hair was dark and wavy, and caught up in loose knots. More could not be seen in the dim light. Presently a man stepped from behind the hangings.

"I have come to say farewell, Mademoiselle Sophie," said he.

He was in full evening dress, his complexion was dark, his features handsome; his brows were level, and his mouth finely-moulded and full. The most remarkable part about him were his eyes—they were dark, and he used them to look straight at all who came within their focus, as if to forestall and prevent a like examination of himself.

"Farewell, Monsieur le Comte," said the girl without moving.

"May I entreat a few moments' conversation with you, Mademoiselle Sophie?"

Her brows contracted, but she simply waved her hand, and said,

"Take a seat, Monsieur le Comte." The way in which she pronounced these words the second time seemed to awaken some suspicion in the man's mind. He drew a chair close to the couch and began—

"Mademoiselle, for the last few days you have avoided me. Have I done anything that has displeased you?"

"Nothing since then," she replied, quietly.

"To what can I attribute this great change in your manner?" he continued in neat incisive tones. "You know I have looked upon you as the one treasure to be sought in life. At first you did not listen unfavourably to my suit, and I even began to hope—"

"Hope nothing whatsoever, Monsieur," said the girl with greater energy than she had used before. "Nor dare to attribute the common politeness shown to one of my brother's friends to other sources than to the hospitality which prompted it."

A dark cloud came over the man's face, not at the words, but at the image they had conjured up. "Your brother has been poisoning your mind against me," said he in a low voice.

This struck fire in the girl.

"My brother?" she said, springing up from the couch, and from the close proximity of those untrustworthy eyes which she had borne so long without flinching. "Do you mention him, whose greatest crime was bringing you to this house, and who has only partially redeemed it, by warning me against you? Not content with winding your toils round a foolish boy, you must needs, mere adventurer though you are, bring a noble family to ruin! Do you dare to sit there, whispering your soft words, and contaminating the very air with your presence? Leave this room, leave this house, instantly, or I will publish to all what a viper lurks in the heart of the family."

"So he told you," said the man in a low tone, and for the first time a shudder passed through her frame. It was so dark now that she could only dimly distinguish his figure as he sat with his head on one hand. She felt the air for the first time hot and oppressive, ; she went quickly to pull up the blind, but when she turned he was gone.

*(To be continued.)*

### Presentation.

The Nurses and Household Staff of the "Whitechapel Infirmary" lately presented Mrs. Perry with an elegant silver salver tea service, and crown Derby cups and saucers as a mark of their affection and esteem. Mrs. Perry has resigned her appointment of matron of this Infirmary, after seven years' service, not as has been said "through ill-health" but from a desire for temporary rest and change of locality.

### Everybody's Opinion.

### PREPARATIONS FOR JUNE.

"One of the First Thousand" writes: In your issue of March 29 appeared a paragraph noting that the first one thousand nurses who joined the Pension Fund were to be presented with certificates by Her Royal Highness the Princess of Wales in June. Will any instructions be given as to where the nurses are to meet, etc., also the date, and would it not be a good plan if some kind friend would undertake to see that provincial nurses, who have no friends in London, were given an address where they could procure comfortable accommodation for one or more nights? [The date and further instructions will be published shortly. Meanwhile all suggestions are valuable.—Editor.]

### NURSE TRAINING SCHOOLS.

A Nightingale Nurse writes: I picked up a small magazine to-day which contained an article giving a list of nurse training schools. Being very proud of being a Nightingale Nurse, I was astonished at the apparent ignorance of the writer of the article, who made no mention of the Nightingale School in connection with St. Thomas's Hospital, but gave undue prominence to several training schools which are little known. On reaching the conclusion of the article, however, I discovered it was written by Mrs. B. Fenwick. Re-reading the list of schools with this clue, I found it was not ignorance, but merely that the writer had carefully excluded all the nurse training schools which signed the great protest against registration. I think this trait in a woman's character is worth recording; it is not often we get such a glimpse of the inner workings of a mind, and, though peculiarly sad to one of my sex, it is also peculiarly instructive. Only I am sorry for the poor Editor who published an article which appears on the face of it to be so incomplete and misleading.

### Appointments.

[It is requested that successful candidates will send a copy of their applications and testimonials, with date of election, to The Editor, The Lodge, Porchester Square, W.]

Blackwell Sanatorium.—Miss F. Gardner, who was for six years a member of the Derby Nursing Association, and afterwards for twelve months staff nurse at Birmingham Hospital has been appointed lady superintendent of the above sanatorium.

Eastbourne Borough Sanatorium.—Miss Ellen Langstaffe, has been appointed head nurse of this sanatorium. Miss Langstaffe holds the certificate of the London Hospital and has worked at the Devon and Exeter Hospitals, and at Dulverton and Bridgwater. We wish Miss Langstaffe all success at her new post.

Fever Hospital, Monsall, Manchester.—We are glad to note that the staff of sisters at this hospital has been increased from four to six. Miss Brown and Miss S. Peter were appointed to fill the vacancies. Miss Brown has been recently night superintendent of the Bristol Royal Infirmary, and Miss Peter night superintendent of the Children's Hospital, Pendlebury, Manchester.

Fulham Infirmary.—Miss A. M. M. Blumenthal was appointed matron of this infirmary on the 10th inst. Miss Blumenthal was trained at Highgate Infirmary, where she served three years. She also holds certificates in midwifery of the Glasgow Maternity Hospital. At the time of her appointment Miss Blumenthal was matron of the Barnhill Hospital, Glasgow, a post she held for four years, and where she won golden opinions from the managers. The ex-chairman testifies that she has excellent administrative capabilities and a clear and comprehensive knowledge of what the sphere of her duties embraces. We congratulate Miss Blumenthal, and wish her every success in her new appointment.

Leeds General Infirmary.—It is with great pleasure we record the appointment of Miss E. R. Miles as assistant superintendent of nurses to Leeds Infirmary. Miss Miles is at present matron of the Hospital for Sick Children, Gateshead-on-Tyne where she has done excellent work.

Madagascar.—Miss Rosa M. Bowesman, sister at the Monsall Fever Hospital, has been appointed matron of the Medical Mission Hospital at Antanànarivo, Madagascar. Miss Bowesman holds the Obstetrical Society's diploma, and was trained at Crumpsall Infirmary.

### Vacancies.

The following vacancies are announced:

### Matrons.

County and County of Carmarthen Infirmary; £40.

District Nurses' Home, Hulme, Manchester; £40.

Dr. Heywood Smith's Hospital.

Exeter Hospital; £60.

Kensington Infirmary (assist.); £40.

Lowestoft Hospital; £50.

### Nurses.

Arbroath Combination Poorhouse; £25.

Abbey Poorhouse; £25.

Aimee Nursing Homes.

Belfast Lying-in Hospital (Midwife); £50.

Beckett Hospital, Barnsley; £24.

Birkenhead Borough Hospital; £25.

Birmingham Skin and Lock Hospital; £10.

Brixton Institute.

Blackheath Nursing Institute.

Blackburn Infirmary; £22.

Cardiff Infirmary.

Carmarthen Infirmary; £23.

Chelsea Infirmary; £18.

Cheltenham Hospital.

City of Lond. Chest Hosp., E.; £22.

Croydon Hospital.

Fitzroy House Home Hospital.

General Nursing Institute.

Greenwich Infirmary; £20.

Gloucester District Nursing Society; £25.

Hanwell Ophthalmic School; £18.

Hartlepools Hospital (night); £25.

Hertford Infirmary (night).

Ipswich Nurses' Home.

King's Cross Hospital, Dundee; £27.

Lewisham Union; £20.

Lowestoft Hospital (night); £20.

Miss Stewart's Home, Glasgow.

Mr. Wilson's Institution.

Newcastle-on-Tyne Infirmary; £20.

Newport Nursing Institute.

North-West London Hospital (night); £24.

Nursing Inst., Newport, I.W.; £20.

Oldham Infirmary; £28.

Peterboro' Infirmary.

Royal Hospital for Children and Women.

Rochdale Infirmary; £22.

St. Mary's Cottage Hospital, Tenbury; £30.

Swansea and South Wales Nursing Institute; £25.

St. Saviour's Infirmary, East Dulwich; £20.

South-Eastern Fever Hospital; £30.

Sheffield Union; £20.

Stephen Monckton Home.

Victoria Hospital, Hull; £20.

Victoria Hospital, Burnley; £26.

West Norfolk and Lynn Hosp.; £20.

West Bromwich Dist. Hosp.; £25.

Whitechapel Infirmary; £20.

West Ham Hospital; £25.

Victoria Home, Bournemouth; £25.

Wakefield Hospital; £25.

Wirral Children's Hospital.

Worthing Infirmary; £24.

### District Nurses.

Ashton-under-Lyne District Nursing Association.

Bristol District Nurses' Home.

Cambridge Home for Nurses.

Cardiff; £30.

Camborne.

Datchet, Windsor.

Lewes; £30.

Maud Hospital, Exmouth.

Nurses' Institute, Jersey; £25.

Tewkesbury; £52.

### Probationers.

Coldstream Cottage Hospital.
Children's Hospital, Sydenham.
Convalescent and Chronic Hospital for Children, N.; fee, £25.
Grimsby Hospital.
Hospital for Women, Liverpool.
Leeds Nurses' Institution; £14.
North-West London Hospital; £8
Stoke-on-Trent Home.
Sheffield Union; £10.
Workhouse Infirmary Nursing Association.
Wisbech Hospital.

### Notes and Queries.

### Queries.

(2) *Waterproof Sheeting.*—Where can I get it good? I have tried all kinds and prices and it always perishes after a few months.—*Matron.*

(3) *Continental Hospitals.*—I should be glad of the names of some which take English nurses.—*Nurse Gertrude.*

### Answers.

*Hospitals and Infirmaries.*—We beg to inform *E. A. C.* that the first title is usually given to public institutions supported by voluntary contributions, the second to institutions supported by Government.

*E. F. W.*—We cannot supply you with the name of the physician.

(3) *Continental Hospitals.*—The Hertford Hospital, Paris, and the Home for Invalid Ladies, San Remo. Perhaps our correspondents can give the names of other institutions.

(74) *F. J.*—Mrs. Craig will be glad to supply this correspondent with information respecting the training she requires for mental nursing, if she will communicate her address to Mrs. Craig, Bishopstone House, Bedford.

### Amusements and Relaxation.

**N.B.—New Word Competition Commenced April 5th, 1890, ends June 28th, 1890.**

N.B.—Word dissections must be sent in WEEKLY to 140, Strand, W.C., arranged alphabetically, with correct total affixed.

The word for dissection for this, the THIRD week of the quarter, being "STANLEY."

| Names. | Ap. 10th. | Totals. |
|---|---|---|
| Adeline | 31 | 31 |
| Patience | 40 | 40 |
| Lightowlers | 38 | 38 |
| Canary | 33 | 33 |
| M. E. S. | 37 | 37 |
| Ambition | 36 | 36 |
| M. W. | 40 | 40 |
| Esperance | 40 | 40 |
| Judy | 37 | 37 |
| Reldas | 39 | 39 |
| Merenda | 29 | 29 |
| Lucy Locket | 32 | 32 |
| Camellia | 30 | 30 |
| Qu'appelle | 40 | 40 |
| Tinie | 40 | 40 |

| Names. | Ap. 10th. | Totals. |
|---|---|---|
| Nurse Mildred | 33 | 33 |
| Coralie | 39 | 39 |
| Gladys | 38 | 38 |
| Agamemnon | 40 | 40 |
| G. E. Y. C. | 24 | 24 |
| S. E. A. | 24 | 24 |
| Jenny Wren | 40 | 40 |
| Multum in parvo | 32 | 32 |
| Ecila | 39 | 39 |
| Weta | 26 | 26 |
| Henri | 35 | 35 |
| Holland | 40 | 40 |
| T. J. | 26 | 26 |
| Nurse Isabel | 17 | 17 |

